# Cardiac Damage Staging in Moderate or Greater Aortic Regurgitation: A New Framework for Risk Stratification

**DOI:** 10.1016/j.shj.2025.100748

**Published:** 2025-10-31

**Authors:** Francisco B. Alexandrino, Ian Persits, Joseph Hajj, Ziad Zalaquett, V. Karthik, Travis Howard, Vincent Chen, Samir R. Kapadia, Serge C. Harb, Rishi Puri

**Affiliations:** a Department of Internal Medicine, Cleveland Clinic, Cleveland, Ohio, USA; b Department of Cardiovascular Medicine, Heart, Vascular, and Thoracic Institute, Cleveland Clinic, Cleveland, Ohio, USA; c NCH Heart Institute, Naples, Florida, USA

**Keywords:** Aortic regurgitation, Aortic valve replacement, Cardiac damage, Global longitudinal strain, Staging

## Abstract

**Background:**

Cardiac damage staging is an established prognostic tool in aortic stenosis, and an initial application to aortic regurgitation (AR) showed prognostic relevance, mainly in advanced stages. No dedicated framework currently exists for AR. We aimed to characterize the extent and prognostic implications of multistructural cardiac damage in patients with moderate or greater AR.

**Methods:**

We retrospectively analyzed 432 adult patients with ≥ moderate AR who underwent transthoracic echocardiography at Cleveland Clinic between 2008 and 2021. Cardiac damage was staged hierarchically from no damage (stage 0) to right ventricular dysfunction (stage 3) based on echocardiographic assessment of left ventricular (LV) global longitudinal strain, LV ejection fraction, chamber dimensions, valvular abnormalities, pulmonary pressures, and right ventricular function. The primary endpoint was a composite of all-cause mortality and heart failure hospitalization.

**Results:**

Over a median follow-up of 5.2 years (interquartile range, 1.9–9.3 years), advancing cardiac damage stage was associated with worsening LV function, progressive atrial and ventricular remodeling, and elevated pulmonary pressures (*p* < 0.001 for all trends). Five-year cumulative incidence of the primary endpoint increased from 3.8% in stage 0 (n = 28), 17.3% in stage 1 (n = 101), 26.2% in stage 2 (n = 210) to 60.7% in stage 3 (n = 93). In multivariable Cox analysis, each stage increment portended a significantly higher risk of adverse outcomes (hazard ratio 2.1 per stage; 95% CI, 1.6–2.6; *p* < 0.0001).

**Conclusions:**

In patients with moderate or greater AR, the burden of cardiac damage incrementally stratified long-term risk. A dedicated staging model specific to AR may refine prognostic assessment and support earlier aortic valve interventions.

## Highlights

• A novel AR-specific cardiac damage staging system was developed and tested.
• Staging integrates GLS with structural and functional echocardiographic markers.
• Progressive cardiac damage was strongly associated with adverse clinical outcomes.

## Graphical abstract

Cardiac damage staging in moderate or greater aortic regurgitation (AR): a new framework for risk stratification. Patients with moderate or severe AR (n = 432) were classified into four hierarchical stages based on progressive echocardiographic evidence of cardiac damage. The Kaplan–Meier curve (right panel) demonstrates a stepwise increase in the composite outcome of all-cause mortality and heart failure hospitalization across stages. Abbreviations: EF, ejection fraction; HF, heart failure; HR, hazard ratio; LV, left ventricle; LA, left atrial; LAVI, left atrial volume index; LVESD, left ventricular end-systolic diameter; MR = mitral regurgitation; GLS, global longitudinal strain; RV, right ventricular; TR, tricuspid regurgitation; RVSP, right ventricular systolic pressure.

**Figure:**
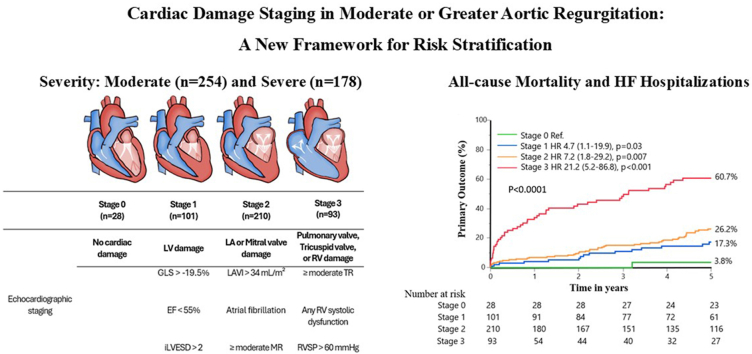

## Introduction

Aortic regurgitation (AR) leads to progressive volume and pressure overload of the left ventricle (LV), prompting compensatory eccentric hypertrophy that maintains systolic function and delays symptom onset.^1^ However, over time, these adaptive mechanisms inevitably fail, leading to rising wall stress, LV dilation, systolic dysfunction, and ultimately heart failure (HF).^1^ Historically, the timing of aortic valve intervention has been guided by the onset of symptoms, reductions in left ventricular ejection fraction (LVEF), or significant LV enlargement based on linear dimensions. Yet, these thresholds are largely derived from studies conducted decades ago.^2^^,^^3^

Recent studies have linked even asymptomatic moderate AR with adverse outcomes, including increased all-cause mortality and HF-related hospitalization rates.^4^, ^5^, ^6^ These findings suggest that the clinical impact of AR likely begins earlier in the disease course than previously thought, precedes conventional echocardiographic thresholds for intervention, occurs in the absence of symptoms, and is linked with heightened mortality.^7^ These observations raise concerns about the adequacy of existing thresholds and highlight the need for more refined risk stratification strategies.^8^^,^^9^ Importantly, the deleterious effects of AR are not confined to the LV. As disease progresses, a pathophysiological cascade involving the left atrium, mitral and tricuspid valves, pulmonary circulation, and right ventricle may ensue.^10^^,^^11^ This continuum of cardiac damage, also influenced by aging and comorbidities, has been well characterized in aortic stenosis, where staging systems based on the extent of cardiac involvement have demonstrated powerful prognostic utility.^12^, ^13^, ^14^ In this context, Silva et al. extrapolated the cardiac damage staging framework from aortic stenosis (AS) to AR in a retrospective cohort. Despite showing the feasibility of this approach, only the most advanced stages (3-4) were independently associated with outcomes and patients classified as stage 0 (no cardiac damage) paradoxically had higher mortality on follow-up than those in stage 1 (LV hypertrophy or LV systolic dysfunction). With this in mind, a reproducible and logical staging system, adjusted to the pathophysiological specificities of AR, has not yet been systematically applied.

Given these evolving insights, a reevaluation of risk stratification strategies in AR is required. In this study, we sought to explore the burden of cardiac damage across multiple cardiac structures in patients with at least moderate AR, utilizing echocardiographic findings, including strain analysis (global longitudinal strain [GLS]), to better characterize disease progression and inform future management strategies.

## Methods

### Study Design and Population

**Figure 1:**
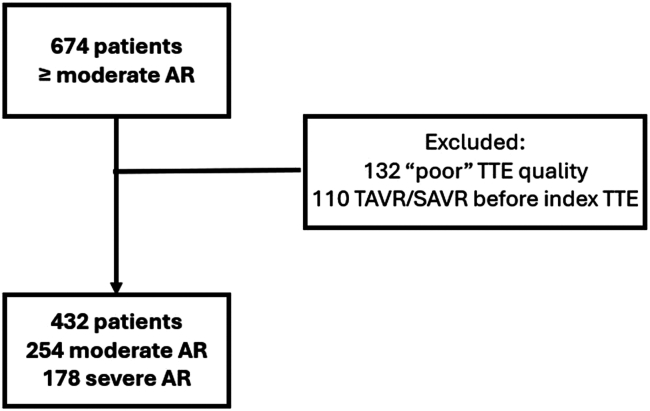
**Study flowchart.** Abbreviation: AR, aortic regurgitation; SAVR, surgical aortic valve regurgitation; TAVR, transcatheter aortic valve replacement; TTE, transthoracic cchocardiogram.

We retrospectively screened patients over the age of 18 years who had an echocardiogram with ≥ moderate AR at Cleveland Clinic from January 2008 through December 2021. AR grading was done by a certified echocardiographer via an integrative approach mostly dependent on qualitative measures. Studies reported as “moderate-to-severe” AR were adjudicated to the severe category for analysis. Studies with poor image quality to obtain GLS measurements were excluded. All other echocardiographic parameters were measurable in these studies, and the exclusion was based solely on inability to assess GLS. Figure 1 summarizes patient selection of the final cohort included in the analyses (*n* = 432). This study was conducted in accordance with the principles outlined in the Declaration of Helsinki. The Cleveland Clinic Institutional Review Board approved this study. The need for written informed consent was waived, as this was a retrospective analysis.

### Definitions

Patients were categorized into four independent stages based on the presence or absence of cardiac damage as assessed by echocardiographic parameters (Table 1), adapted from prior validation in aortic stenosis.^13^ Stage 0 was defined as the absence of detectable cardiac damage. Stage 1 reflected LV damage, characterized by a GLS greater than −19.5%, a cutoff associated with worse survival in chronic asymptomatic AR,^15^ a LVEF less than 55%, or an indexed left ventricular end-systolic diameter (LVESD) greater than 2.^16^ Stage 2 encompassed left atrial (LA) or mitral valve damage, defined by a LA volume index greater than 34 mL/m^2^, the presence of atrial fibrillation, or moderate or greater mitral regurgitation. Stage 3 indicated more advanced structural involvement, including pulmonary hypertension (right ventricular [RV] systolic pressure >60 mmHg), moderate or greater tricuspid regurgitation (TR), or any degree of RV systolic dysfunction.

**Table 1:** Staging definitions

	Stage 0 (n = 28)	Stage 1 (n = 101)	Stage 2 (n = 210)	Stage 3 (n = 93)
	No cardiac damage	LV damage	LA or mitral valve damage	Pulmonary valve, tricuspid valve, or RV damage
Echocardiographic staging		GLS >-19.5%	LAVI >34 mL/m^2^	≥ moderate TR
		EF <55%	Atrial fibrillation	Any RV systolic dysfunction
		iLVESD >2	≥ moderate MR	RVSP >60 mmHg

Abbreviations: EF, ejection fraction; GLS, global longitudinal strain; iLVESD, indexed left ventricular end-systolic diameter; LA, left atrial; LAVI, left atrial volume index; LV, left ventricle; MR, mitral regurgitation; RV, right ventricular; RVSP, right ventricular systolic pressure; TR, tricuspid regurgitation.

Patients were hierarchically classified to the highest stage if at least one of the proposed criteria was met. These criteria were selected based on current echocardiography guidelines and supported by contemporary studies validating the selected thresholds.^15^^,^^16^ All transthoracic echocardiograms were performed and reviewed at the Cleveland Clinic echocardiography laboratory by experienced sonographers, using standardized acquisition protocols in accordance with the guidelines.^17^ GLS and LA volumes were measured retrospectively by trained physicians blinded to clinical outcomes. Other baseline and echocardiographic variables, including the presence of bicuspid aortic valve and concomitant mitral or tricuspid valve disease, were systematically collected from electronic medical records and echocardiography reports; when relevant, image re-review was performed to confirm key findings. Comorbidities, including atrial fibrillation, HF, chronic kidney disease (CKD), coronary artery disease (CAD), hypertension, stroke, chronic obstructive pulmonary disease, diabetes mellitus, and presence of pacemaker, were extracted using International Classification of Diseases, 10th Revision (ICD-10) diagnosis codes with cross-checking against chart review when necessary to maximize accuracy. N-terminal pro-B-type natriuretic peptide (NT pro-BNP) levels were extracted if present within 6 months of index transthoracic echocardiogram (TTE).

### Study Outcomes

The primary outcome was a composite of all-cause mortality and hospitalization for HF during follow-up. Hospitalizations for HF were first identified based on ICD coding and confirmed based on clinical criteria such as signs and symptoms of congestion and/or requirement of diuresis. Outcomes were identified through detailed review of the electronic medical records. Patients who underwent transcatheter aortic valve replacement (TAVR) or surgical aortic valve replacement (SAVR) during follow-up were censored at the time of the procedure.

### Statistical Analysis

Continuous data are presented as median ± interquartile range (IQR) and were compared between groups using the Student’s *t*-test or the Wilcoxon rank sum test, as appropriate. Categorical variables were presented as count and percent and compared using the χ^2^ or the Fisher exact test. We estimate time-to-event data using Kaplan–Meier techniques. Cox proportional hazard regression analyses were performed to assess the association between staging classification or other clinical parameters with the primary outcome. The Cox proportional hazard model was adjusted for variables statistically significant in the univariable analysis or considered clinically relevant. All statistical analyses were performed using JMP version 18.0 (SAS Institute Inc, Cary, NC).

## Results

### Study Population

A total of 432 patients with ≥ moderate AR between 2008 and 2021 were followed for a median time of 5.2 years (IQR, 1.9–9.3 years). Fifty-nine percent (n = 254) of the population had moderate AR, and severe AR was more prevalent (53.7%) in stage 3 when compared to the other stages (*p* = 0.048) (Supplementary Table S2). At the time of the index echocardiogram, 28 patients (6.5%) were classified as stage 0 (no cardiac damage), 101 (23.4%) as stage 1 (left ventricular damage), 210 (48.6%) as stage 2 (LA or mitral valve damage), and 93 (21.5%) as stage 3 (pulmonary valve, tricuspid valve, or RV damage). Table 2 summarizes the rates of each individual cardiac damage component within each stage. Patients classified in higher stages were older (*p* < 0.0001) and exhibited a higher prevalence of comorbidities, including atrial fibrillation, HF, CKD, CAD, hypertension, prior pacemaker implantation, and diabetes mellitus, as detailed in Table 3. Fifty-four patients (12.5%) underwent TAVR or SAVR within a median of 6.9 years (IQR, 3.9-10.1) from the index echocardiogram.

**Table 2:** Rates of each individual cardiac damage component within each stage

	Stage 0 (n = 28)	Stage 1 (n = 101)	Stage 2 (n = 210)	Stage 3 (n = 93)
GLS >-19.5%	0 (0%)	94 (93.1%)	164 (78.1%)	82 (88.2%)
iLVESD >2	0 (0%)	15 (14.9%)	43 (20.5%)	26 (28.0%)
LVEF <55%	0 (0%)	23 (22.8%)	42 (20%)	40 (43.0%)
LAVI >34	0 (0%)	0 (0%)	135 (64.3%)	71 (76.3%)
Atrial fibrillation	0 (0%)	0 (0%)	155 (73.8%)	72 (77.4%)
≥ moderate MR	0 (0%)	0 (0%)	37 (17.6%)	29 (31.2%)
RVSP >60 mmHg	0 (0%)	0 (0%)	0 (0%)	21 (22.6%)
≥ moderate TR	0 (0%)	0 (0%)	0 (0%)	75 (80.6%)
RV systolic dysfunction	0 (0%)	0 (0%)	0 (0%)	36 (38.7%)

Abbreviations: GLS, global longitudinal strain; iLVESD, indexed left ventricular end-systolic diameter; LAVI, left atrial volume index; LVEF, left ventricular ejection fraction; MR, mitral regurgitation; RV, right ventricular; RVSP, right ventricular systolic pressure; TR, tricuspid regurgitation.

**Table 3:** Baseline characteristics

	Stage 0 (n = 28)	Stage 1 (n = 101)	Stage 2 (n = 210)	Stage 3 (n = 93)	*p* value
Female sex	11 (39.3%)	50 (49.5%)	85 (40.5%)	46 (49.5%)	0.3034
Age (years)	56.5 (43-68)	63 (50.5-74.0)	68.0 (58.0-76.0)	74.0 (63.0-82.5)	<0.0001
African-American	0 (0%)	13 (12.9%)	23 (11.0%)	10 (10.8%)	0.6622
BMI	25.8 (24.0-27.2)	27.8 (24.8-30.7)	27.5 (24.3-31.3)	26.8 (24.0-31.8)	0.4355
NT pro BNP (pg/mL)	76 ± 11	1293 ± 3398	4852 ± 1755	8934 ± 1682	0.14
Atrial fibrillation	0 (0%)	0 (0%)	55 (26.2%)	21 (22.6%)	<0.0001
Severe AR	11 (39.3%)	36 (35.6%)	81 (38.6%)	50 (53.7%)	0.048
HF	9 (32.1%)	49 (48.5%)	140 (66.7%)	82 (88.2%)	<0.0001
CKD	3 (10.7%)	23 (22.8%)	64 (30.5%)	38 (40.9%)	0.0051
CABG	4 (14.3%)	12 (11.9%)	47 (22.4%)	18 (19.4%)	0.1489
CAD	11 (39.3%)	50 (49.5%)	137 (65.2%)	57 (61.3%)	0.0079
Hypertension	18 (64.3%)	85 (84.2%)	192 (91.4%)	75 (80.7%)	0.0004
Pacemaker	1 (3.6%)	6 (5.9%)	52 (24.8%)	29 (31.2%)	<0.0001
Stroke	8 (28.6%)	28 (27.7%)	91 (43.3%)	33 (35.5%)	0.0405
COPD	5 (17.9%)	35 (34.7%)	65 (31.0%)	38 (40.9%)	0.1109
DM	3 (10.7%)	24 (23.8%)	54 (25.7%)	24 (25.8%)	0.3665

Abbreviations: AR, aortic regurgitation; BMI, body mass index; CABG, coronary artery bypass grafting; CAD, coronary artery disease; CKD, chronic kidney disease; COPD, chronic obstructive pulmonary disease; DM, diabetes mellitus; HF, heart failure; NT pro BNP, N-terminal pro-B type natriuretic peptide.

Table 4 summarizes the echocardiographic findings showing the hierarchical nature of the staging system, demonstrating worsening ventricular function, atrial remodeling, and valvular dysfunction across advancing stages of cardiac damage. The prevalence of bicuspid aortic valve morphology decreased significantly across stages, from 53.6% in stage 0 to 7.5% in stage 3 (*p* < 0.0001). Measures of left ventricular structure and function progressively worsened with increasing stage severity. GLS declined from a median of −20.7% (IQR, −22.8 to −20.1) in stage 0 to −12.2% (IQR, −16.7 to −8.0) in stage 3 (*p* < 0.0001), while LVEF dropped from 61.9% (IQR, 60.0–65.0) to 55.0% (IQR, 45.0–60.0) (*p* < 0.0001). Importantly, in our reproducibility analysis (n = 20), interobserver correlation for GLS was strong (r = 0.95; 95% CI, 0.86–0.98), with a mean absolute difference of 1.4% and Root Mean Square Error (RMSE) of 1.6%. LVESD increased from 2.8 cm (IQR, 2.6–3.0) in stage 0 to 3.3 cm (IQR, 2.8–4.0) in stage 3 (*p* = 0.0003). Markers of LA remodeling also showed stepwise worsening across stages. LA volume index increased from 24.7 mL/m^2^ (IQR, 20.4–30.8) in stage 0 to 46.6 mL/m^2^ (IQR, 35.4–58.5) in stage 3 (*p* < 0.0001), accompanied by progressive increases in LA diameter and volume. Mitral and tricuspid valve disease were increasingly prevalent: moderate or greater mitral regurgitation was observed in 17.6% of patients in stage 2 and 31.2% in stage 3 (*p* < 0.0001), while moderate or greater TR was present in 80.7% of stage 3 patients and absent in all others (*p* < 0.0001). RV systolic pressure rose substantially with increasing stage, from 26.5 mmHg (IQR, 23.0–30.1) in stage 0 to 45.5 mmHg (IQR, 35.8–56.9) in stage 3 (*p* < 0.0001), consistent with progressive pulmonary hypertension. Additional echocardiographic indicators of diastolic dysfunction, such as mitral valve (MV) E/e′ lateral and septal, also increased markedly across stages (both *p* < 0.0001). The prevalence of diastolic dysfunction, defined as MV E/e′ septal >14, increased progressively with advancing stage (stage 0: 15.8%, stage 1: 31.3%, stage 2: 54.4%, stage 3: 74.3%; *p* < 0.0001).

**Table 4:** Echocardiographic characteristics

	Stage 0 (n = 28)	Stage 1 (n = 101)	Stage 2 (n = 210)	Stage 3 (n = 93)	*p* value
LVEF (%)	61.9 (60.0-65.0)	60.0 (55.0-62.5)	59.1 (55.0-62.7)	55.0 (45.0-60.0)	<0.0001
RVSP (mmHg)	26.5 (23.0-30.1)	28.0 (24.0-33.9)	30.3 (27.1-37.7)	45.5 (35.8-56.9)	<0.0001
AV area (cm^2^)	1.7 (1.4-1.8)	1.4 (1.2-1.7)	1.4 (1.2-1.7)	1.4 (1.2-1.5)	0.0346
AV mean gradient (mmHg)	15.3 (13.0-21.7)	15.0 (10.8-19.4)	16.0 (12.0-20.0)	12.7 (10.0-17.4)	0.0674
AV peak gradient (mmHg)	30.0 (24.0-36.0)	27.8 (19.9-34.1)	28.9 (21.5-36.6)	22.2 (15.8-32.4)	0.0250
AV max velocity (m/s)	2.7 (2.4-3.0)	2.6 (2.3-2.9)	2.8 (2.4-3.1)	2.3 (1.9-2.8)	0.0004
Bicuspid	15 (53.6%)	31 (30.7%)	43 (20.5%)	7 (7.5%)	<0.0001
LA diameter (cm)	3.8 (3.3-4.1)	3.6 (3.3-4.0)	4.2 (3.6-4.7)	4.7 (4.1-5.3)	<0.0001
LA volume (mL)	44.0 (35.8-61.8)	49.2 (38.9-59.1)	71.9 (58.1-92.7)	92.0 (60.5-120.7)	<0.0001
LAVI (mL/m^2^)	24.7 (20.4-30.8)	25.7 (21.1-29.4)	36.7 (29.0-46.9)	46.6 (35.4-58.5)	<0.0001
GLS endocardium (%)	-20.7 (-22.8- -20.1)	-16 (-18.5- -13.7)	-15.7 (-19- -12.8)	-12.2 (-16.7- -8.0)	<0.0001
LVEDD (cm)	4.7 (4.4-5.0)	4.7 (4.2-5.3)	5.0 (4.4-5.5)	4.7 (4.3-5.4)	0.1001
LVEDV (mL)	113.0 (87.0-130.0)	100.1 (80.1-122.4)	120.9 (86.1-155.0)	106.4 (69.8-134.7)	0.0062
LVESD (cm)	2.8 (2.6-3.0)	3.0 (2.6-3.5)	3.2 (2.7-3.7)	3.3 (2.8-4.0)	0.0003
LVESV (mL)	41.0 (30.2-46.6)	40.9 (31.1-56.3)	47.0 (33.9-67.4)	44.9 (27.4-72.3)	0.0265
>=mod MR	0 (0%)	0 (0%)	37 (17.6%)	29 (31.2%)	<0.0001
> = mod TR	0 (0%)	0 (0%)	0 (0%)	75 (80.7%)	<0.0001
MV E/e’ lateral	8.0 (7.2-11.3)	8.2 (6.8-11.6)	11.3 (7.9-17.9)	13.6 (9.9-20.5)	<0.0001
MV E/e’ septal	9.5 (8.5-12.4)	11.9 (9.0-15.9)	14.6 (11.0-20.6)	16.9 (13.4-36.0)	<0.0001

Abbreviations: AV, aortic valve; GLS, global longitudinal strain; LA, left atrial; LAVI, left atrial volume index; LVEF, left ventricular ejection fraction; LVEDD, left ventricular end-dastolic diameter; LVEDV, left ventricular end-diastolic volume; LVESD, left ventricular end-systolic diameter; LVESV, left ventricular end-systolic volume; MR, mitral regurgitation; MV, mitral valve; RVSP, right ventricular systolic pressure; TR, tricuspid regurgitation.

### Outcomes and Associated Risk Factors

**Figure 2:**
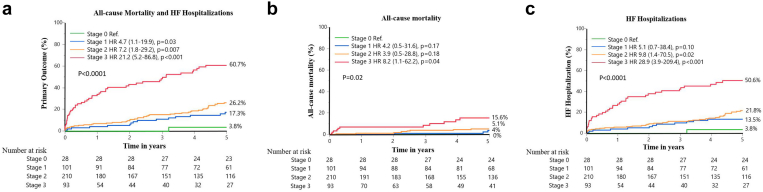
**Kaplan–Meier survival curves according to cardiac damage stage.** (a) Cumulative incidence of the primary composite endpoint (all-cause mortality or heart failure hospitalization) across cardiac damage stages. (b) All-cause mortality. (c) Heart failure hospitalization. Abbreviations: HF, heart failure; HR, hazard ratio.

Over a median follow-up of 5.2 years, clinical outcomes worsened progressively across higher stages of cardiac damage. After censoring patients who underwent TAVR or SAVR during follow-up, the cumulative incidence of the primary outcome, composite of all-cause mortality or HF hospitalization at 5 years, was 3.8% in stage 0, 17.3% in stage 1, 26.2% in stage 2, and 60.7% in stage 3 (Figure 2a-c). Across the entire study duration, event rates remained strongly associated with stage severity at different time points (1, 3, and 5 years). At 5 years, all-cause mortality increased progressively across stages, from 0% in stage 0 to 15.6% in stage 3, while HF hospitalizations showed a much steeper rise from 3.8% in stage 0 to 50.6% in stage 3 (Table 5).

**Table 5:** Cumulative incidence of events at 1, 3, and 5 years for each disease stage

	Stage 0 (n = 28)	Stage 1 (n = 101)	Stage 2 (n = 210)	Stage 3 (n = 93)
Composite endpoint	1 y: 0% 3 y: 0% 5 y: 3.8%	1 y: 4.2% 3 y: 11% 5 y: 17.3%	1 y: 7% 3 y: 15.2% 5 y: 26.2%	1 y: 34% 3 y: 48.4% 5 y: 60.7%
All-cause mortality	1 y: 0% 3 y: 0% 5 y: 0%	1 y: 0% 3 y: 1.2% 5 y: 4%	1 y: 1% 3 y: 3.8% 5 y: 5.1%	1 y: 7.1% 3 y: 8.7% 5 y: 15.6%
Heart failure hospitalization	1 y: 0% 3 y: 0% 5 y: 3.8%	1 y: 4.2% 3 y: 9.9% 5 y: 13.5%	1 y: 6% 3 y: 11.5% 5 y: 21.8%	1 y: 28.1% 3 y: 42.3% 5 y: 50.6%

Outcomes are shown separately by the composite endpoint, all-cause mortality, and heart failure hospitalization.

Compared with stage 0, the risk of the primary outcome increased progressively with higher stages of cardiac damage. Patients in stage 1 had a hazard ratio (HR) of 4.7 (95% CI, 1.1–19.9; *p* = 0.03), those in stage 2 had an HR of 7.2 (95% CI, 1.8–29.2; *p* = 0.007), and those in stage 3 had the highest risk, with an HR of 21.2 (95% CI, 5.2–86.8; *p* < 0.001).

Cox proportional hazards regression analysis was performed in the overall cohort (Table 6). In unadjusted analyses, higher stage classification, older age, HF, CKD, coronary artery bypass grafting, CAD, atrial fibrillation, pacemaker implantation, prior stroke, chronic obstructive pulmonary disease, and diabetes were each significantly associated with increased risk of death. After multivariable adjustment, advancing cardiac damage stage (continuous variable) remained independently associated with all-cause mortality (HR, 2.1 per stage increase; 95% CI, 1.6–2.6; *p* < 0.0001). Other independent associated factors with mortality included older age (HR, 1.05 per year; 95% CI, 1.03–1.07; *p* < 0.0001), history of HF (HR, 2.0; 95% CI, 1.3–3.1; *p* < 0.01), prior coronary artery bypass grafting (HR, 1.8; 95% CI, 1.2–2.6; *p* < 0.01), and diabetes mellitus (HR, 1.9; 95% CI, 1.4–2.7; *p* < 0.01).

**Table 6:** Univariable and multivariable cox proportional hazard regression

Variable	Univariate		Multivariate	
	HR (95% CI)	*p* value	HR (95% CI)	*p* value
Stage (per each level increase)	2.3 (1.9-2.9)	<0.01	2.1 (1.6-2.6)	**<0.01**
Female	1.3 (0.98-1.8)	0.06		
Age (per each y increase)	1.06 (1.04-1.08)	<0.01	1.05 (1.03-1.07)	**<0.01**
Atrial fibrillation	2.1 (1.5-2.8)	<0.01	0.84 (0.58-1.2)	0.28
HF	3.9 (2.7-5.8)	<0.01	2.0 (1.3-3.1)	**<0.01**
CKD	2.6 (1.9-3.5)	<0.01	1.2 (0.82-1.6)	0.36
Prior CABG	2.1 (1.5-3.0)	<0.01	1.8 (1.2-2.6)	**<0.01**
CAD	1.6 (1.2-2.2)	<0.01	0.82 (0.58-1.2)	0.29
Hypertension	1.5 (0.88-2.5)	0.12		
Pacemaker	2.0 (1.4-2.7)	<0.01	1.2 (0.83-1.4)	0.40
Stroke	1.7 (1.3-2.3)	<0.01	0.98 (0.69-1.4)	0.90
COPD	2.0 (1.5-2.7)	<0.01	1.3 (0.92-1.7)	0.13
DM	2.3 (1.7-3.2)	<0.01	1.9 (1.4-2.7)	**<0.01**

Variables included in the analysis were selected based on clinical relevance. In the multivariate model, only variables that were statistically significant in the univariate analysis were retained.

Abbreviations: CABG, coronary artery bypass grafting; CAD, coronary artery disease; CKD, chronic kidney disease; COPD, chronic obstructive pulmonary disease; DM, diabetes mellitus; HF, heart failure; HR, hazard ratio.

A sensitivity analysis further demonstrated that even early LV damage, defined solely by impaired GLS (>-19.5%), was independently associated with worse outcomes compared to stage 0 (HR, 4.7; 95% CI, 1.1–20.0; *p* = 0.04). For simplicity and to maintain adequate statistical power, early (n = 67) and late (n = 34) LV damage were ultimately combined into a single stage 1 category in the final staging model. This stratification allowed us to isolate the prognostic role of GLS in the absence of other cardiac damage markers. Only 7 patients (20%) in the late LV damage subgroup had normal GLS with abnormal LVEF and/or indexed LVESD. Applying the same principle to stage 3, which included right-sided involvement, we separated patients with isolated tricuspid/pulmonary damage from those with RV dysfunction and found that RV dysfunction conferred additional risk (HR, 2.1; 95% CI, 1.2–3.4; *p* = 0.005) (Supplementary Material).

To further examine the influence of age and sex on the prognostic value of our prespecified GLS cutoff of −19.5%, we conducted sensitivity analyses stratified by these subgroups. P interaction between GLS and both age and sex was not significant (*p* = 0.72 and *p* = 0.13) (Supplementary Material).

## Discussion

In this large cohort of patients with moderate or greater AR, we developed a novel cardiac damage staging system specifically tailored to the unique pathophysiology of AR and performed internal validation within a single-center cohort (Graphical Abstract). Our findings demonstrate that progressive multistructural cardiac damage, characterized by early LV dysfunction, atrial and ventricular remodeling, pulmonary hypertension, and eventual RV dysfunction, was strongly and incrementally associated with adverse clinical outcomes. Importantly, this staged model, adapted from validated aortic stenosis cohorts,^13^ incorporates additional novel parameters such as GLS and provides robust prognostic stratification for an AR population even after adjustment for traditional risk factors including age.

The concept of staging cardiac damage was initially proposed in the context of AS, where the extent of extravalvular cardiac injury was found to be associated with mortality independently of valve hemodynamics. While this framework has subsequently been applied to AR,^18^ these adaptations did not account for the distinct remodeling processes inherent to chronic volume overload conditions. Unlike the pressure-overload state of AS, AR results in chronic volume overload, leading first to compensatory chamber dilation and eccentric hypertrophy rather than concentric remodeling.^2^ Consequently, conventional echocardiographic parameters that guide surgical/interventional planning in asymptomatic patients with AR often underestimate the degree of myocardial damage in these patients, which may delay crucial interventions.^2^, ^3^, ^4^ Importantly, our study also uniquely included patients with moderate AR, allowing for earlier detection of cardiac changes before overt valvular deterioration occurs, further enhancing the clinical applicability of this staging system.

Our proposed staging system addresses this gap by incorporating GLS, a sensitive marker of early myocardial dysfunction that precedes reductions in LV ejection fraction. Prior studies have established the prognostic utility of GLS in AR,^5^ and our results further reinforce its value in capturing early stages of cardiac decompensation. Age and sex are known to influence normal GLS values,^19^ but in our sensitivity analyses, no significant interactions were observed between these variables and GLS.

Notably, our staging approach avoided the paradoxical finding observed in prior adaptations,^18^ where stage 1 patients (with isolated LA or LV dilatation) exhibited worse outcomes than stage 0 patients, suggesting a more coherent biological gradient of disease severity when GLS is included. This was reflected in the progressive increase in HRs across stages, with stage 1 already associated with a four-fold higher risk of adverse outcomes (HR, 4.7; 95% CI, 1.1–20.0; *p* = 0.04) compared to stage 0, and risk magnifying substantially through intermediate and advanced stages (HR, 21.2 for stage 3; 95% CI, 5.2–86.8; *p* < 0.001). Also, sensitivity analyses further demonstrated that GLS alone carried prognostic value in the absence of other cardiac damage markers, providing mechanistic insight into the stepwise progression of LV remodeling. This is particularly relevant for patients who are asymptomatic or have lower NT-proBNP levels (masked by obesity), in whom early identification of risk is most challenging. Of note, HF hospitalizations were the principal drivers of adverse outcomes, more so than mortality, emphasizing the importance of evaluating composite endpoints rather than crude mortality alone. This contrasts with prior adaptations of the AS staging framework to AR, which focused only on mortality, whereas our study highlights HF hospitalizations as the main driver of risk—which we feel is far more clinically relevant as it is HF encounters which we typically manage to prevent in day-to-day clinical practice. This distinction makes clinicians more aware of the practical utility of the staging system as a tool to anticipate and potentially prevent rehospitalizations. By incorporating GLS and demonstrating a coherent biological gradient, our model strengthens clinical applicability and underscores its potential role in improving patient outcomes. The coexistence of multiple valvular lesions is not uncommon, with mitral regurgitation (MR) and TR present in 15 and 17% of our cohort, respectively, rates consistent with prior literature.^10^ Both MR and TR were associated with significantly increased mortality, underscoring their role as markers of advanced disease and ventricular-atrial remodeling. These findings highlight the importance of recognizing concomitant valvular dysfunction in chronic AR, as it may identify patients who have already transitioned to a higher-risk stage despite preserved ejection fraction, and support their incorporation into clinical staging algorithms to improve risk stratification.

Age emerged as a strong association with mortality, as expected, with patients in higher stages tending to be older. Besides this, patients in higher stages also had a higher comorbidity burden, namely atrial fibrillation, HF, CKD, CAD, and hypertension, in line with what is seen in prior staging systems, either in MR,^20^ AR,^18^ or AS.^13^ A recent study showed that nearly 80% of patients with mild AS had some degree of cardiac damage, with higher stages strongly linked to comorbidities.^21^ Our findings are aligned in that older age and comorbidities were more prevalent in advanced stages; however, we observed a lower proportion of patients without cardiac damage (6%), and importantly, severe AR was more frequent in stage 3, despite not being associated with worse outcomes in isolation. This suggests that, unlike mild AS, the progression of AR itself contributes significantly to cardiac damage and adverse outcomes, beyond the effect of age and comorbidities alone. Future studies assessing cardiac damage in patients with mild AR would be valuable in clarifying this question. The staging model remained a powerful independent association with adverse outcomes even after multivariable adjustment, highlighting the dominant prognostic role of structural cardiac damage over chronological age and all comorbidities. We recognize older age and higher comorbidity prevalence is a potential source of bias that cannot be fixed due to the study nature; however, the purpose of the study is to risk stratify moderate AR and not to attribute sole causality to it.

Interestingly, patients with bicuspid aortic valves were more commonly found in the earlier stages of cardiac damage. This observation aligns with previous reports suggesting that AR associated with bicuspid valves may develop earlier in life, at a time when myocardial reserve is greater and the adaptive remodeling capacity is higher.^22^ This potentially more benign remodeling response may partly explain the slower progression to advanced cardiac damage observed in these patients compared to those with degenerative or inflammatory etiologies of AR.

A key strength of the present validation study lies in the novel, AR-specific staging framework, which recognizes the unique pathophysiology of chronic volume overload by integrating strain imaging with traditional echocardiographic parameters. Our large, well-phenotyped cohort with long-term clinical follow-up allowed for robust evaluation of the prognostic value of the proposed staging model. Additionally, the use of routinely obtainable and reproducible echocardiographic measures enhances the feasibility of clinical implementation. Our inclusion of moderate AR patients revealed that a subset already had stage 2 or 3 cardiac damage, emphasizing the value of staging as a surveillance tool in this population, as it is already recognized as being associated with poor prognosis.^23^^,^^24^ With the results of the recent Transcatheter Aortic Valve Implantation in Patients with High-Risk Syptomatic Native Aortic Regurgitation (ALIGN-AR) trial,^25^ we anticipate that TAVR will be increasingly applied in patients with isolated symptomatic moderate-severe AR at high surgical risk, making the incorporation of cardiac damage staging valuable in this population. Pivotal trials managing moderate AS with TAVR have completed enrollment and Cardiovascular Outcomes Assessment of the MitraClip Percutaneous Therapy for Heart Failure Patients with Functional Mitral Regurgitation (COAPT-2) trial is starting recruitment in moderate functional MR using MitraClip. While we tend to think of valvular disease severity as stages in isolation, it is clear that cardiac damage accrues as a continuum, and multiple moderate valve lesions likely harbor significant cardiac damage, morbidity, and mortality. Yang et al. showed in a large cohort of patients with moderate-severe AR that the presence of ≥ MR doubles the risk of mortality, and the presence of triple valvular disease with ≥ moderate TR quadruples this risk.^10^ The prevalence of ≥ moderate MR and TR in that study (14 and 12%, respectively) closely parallels our present findings (15 and 17%). This suggests that mixed valvular disease represents a marker of greater disease severity, and cardiac damage staging is a pragmatic and reproducible framework to capture this continuum of risk. Further prospective studies and trials are needed to determine whether staging can refine the timing of aortic valve replacement (AVR), especially in moderate AR, and capture structural recovery after intervention.

Several limitations of the present analysis should be considered. First, its retrospective design precludes causal inference, and it remains uncertain whether the observed stages of damage are directly attributable to AR or represent coexisting myocardial pathology. Second, RV dysfunction was assessed visually, without the use of quantitative measures such as tricuspid annular plane systolic excursion or RV strain, which is less specific and may introduce interobserver variability. However, visual assessment remains the most commonly used method in routine clinical practice and allows pragmatic, real-world identification of patients with advanced disease, particularly those at highest risk. Third, while GLS offers significant prognostic information, its use remains variable across institutions, and technical factors such as vendor differences and interobserver variability may affect its measurement.^5^ Finally, external validation in independent cohorts will be necessary to confirm the generalizability of our findings for clinical practice.

## Conclusion

Our results suggest that comprehensive assessment of cardiac damage, beyond traditional metrics of valve severity and LV ejection fraction, may improve risk stratification in patients with at least moderate AR. Early identification of subclinical myocardial dysfunction using GLS and recognition of progressive multistructural damage could help guide timing of aortic valve intervention before irreversible myocardial injury ensues. Incorporation of this staging model into clinical practice and prospective studies evaluating its utility in surgical decision-making are warranted.

## Ethics Statement

This study was conducted in accordance with the principles outlined in the Declaration of Helsinki. The research protocol was reviewed and approved by the Cleveland Clinic Institutional Review Board. Given the retrospective nature of the study and the use of de-identified data, the requirement for written informed consent was waived.

## Disclosure Statement

The authors report no conflict of interest.
