# Supplementary material for: Cardiac Damage Staging in Moderate or Greater Aortic Regurgitation: A New Framework for Risk Stratification
Source: Struct Heart. 2025 Oct 31;10(1):100748. doi: 10.1016/j.shj.2025.100748 (PMC12686691; doi:10.1016/j.shj.2025.100748)

**Supp. Table 1. Staging definitions**

|  | **Stage 0**  **(n=28)** | **Stage 1**  **(n=67)** | **Stage 2**  **(n=34)** | **Stage 3**  **(n=210)** | **Stage 4**  **(n=57)** | **Stage 5**  **(n=36)** |
| --- | --- | --- | --- | --- | --- | --- |
| Echocardiographic staging | **No cardiac damage** | **Early**  **LV damage** | **Late**  **LV damage** | **LA or Mitral damage** | **Pulmonary or Tricuspid damage** | **RV damage** |
|  |  | GLS > -19.5% | iLVESD >2  EF <55% | LAVI >34 mL/m² | ≥ moderate TR  RVSP >60 mmHg |  |
|  |  |  |  | Atrial  fibrillation |  | Any RV systolic dysfunction |
|  |  |  |  | ≥ moderate MR |  |  |

**Supp Table 2. Rates of each stages by aortic regurgitation severity**

|  | **Stage 0**  **(n=28)** | **Stage 1**  **(n=101)** | **Stage 2**  **(n=210)** | **Stage 3**  **(n=93)** | **p-value** |
| --- | --- | --- | --- | --- | --- |
| Moderate AR (%) | 17 (60.7) | 65 (64.4) | 129 (61.4) | 43 (46.2) | 0.048 |
| Severe AR (%) | 11 (39.3) | 36 (35.6) | 81 (38.6) | 50 (53.8) |  |

**Supp. Table 3. Rates of Each Individual Cardiac Damage Component Within Each Stage**

|  | **Stage 0**  **(n=28)** | **Stage 1**  **(n=67)** | **Stage 2**  **(n=34)** | **Stage 3**  **(n=210)** | **Stage 4**  **(n=57)** | **Stage 5**  **(n=36)** |
| --- | --- | --- | --- | --- | --- | --- |
| GLS > -19.5% | 0 (0%) | 67 (100%) | 27 (79.4%) | 164 (78.1%) | 48 (84.2%) | 34 (94.4%) |
| iLVESD > 2 | 0 (0%) | 0 (0%) | 15 (44.1%) | 43 (20.5%) | 13 (22.8%) | 13 (36.1%) |
| LVEF < 55% | 0 (0%) | 0 (0%) | 23 (67.7%) | 42 (20%) | 16 (28.1%) | 24 (66.7%) |
| LAVI > 34 | 0 (0%) | 0 (0%) | 0 (0%) | 135 (64.3%) | 45 (79.0%) | 26 (72.2%) |
| Atrial fibrillation | 0 (0%) | 0 (0%) | 0 (0%) | 155 (73.8%) | 43 (75.4%) | 29 (80.6%) |
| ≥ moderate MR | 0 (0%) | 0 (0%) | 0 (0%) | 37 (17.6%) | 17 (29.8%) | 12 (33.3%) |
| RVSP > 60 mmHg | 0 (0%) | 0 (0%) | 0 (0%) | 0 (0%) | 15 (26.3%) | 6 (16.7%) |
| ≥ moderate TR | 0 (0%) | 0 (0%) | 0 (0%) | 0 (0%) | 52 (91.2%) | 23 (63.9%) |
| RV systolic dysfunction | 0 (0%) | 0 (0%) | 0 (0%) | 0 (0%) | 0 (0%) | 36 (100%) |

**Supp Table 4. NT pro-BNP by stage**

|  | **Stage 0**  **(n=28)** | **Stage 1**  **(n=101)** | **Stage 2**  **(n=210)** | **Stage 3**  **(n=93)** | **p-value** |
| --- | --- | --- | --- | --- | --- |
| NT pro-BNP (pg/mL)  [% available] | 76  [4] | 1292.5 (63-2521)  [12] | 4852 (1307-8397)  [21] | 8934 (5201-12665)  [53] | 0.14 |
|  |  |  |  |  |  |

**Sensitivity analysis of GLS with age and sex**

To further examine the influence of age and sex on the prognostic value of our prespecified GLS cutoff of −19.5%, we conducted sensitivity analyses stratified by these subgroups. In sex-stratified analyses, mean GLS was similar between males (−15.1% [95% CI −15.8 to −14.5]) and females (−15.3% [−16.0 to −14.6]; p = 0.76). Using the prespecified cutoff of −19.5%, impaired GLS was strongly associated with outcomes in males (HR 4.9, 95% CI 2.27–10.69) but not in females (HR 1.5, 95% CI 0.82–2.91). However, the GLS × sex interaction was not significant (p interaction = 0.72), indicating no statistical evidence that sex modified the prognostic effect of GLS. Similarly, patients <65 years had more favorable GLS values on average (−16.2% [−16.9 to −15.5]) compared with those ≥65 years (−14.5% [−15.1 to −13.9]; p=0.004). Impaired GLS predicted adverse outcomes in younger patients (HR 16.4, 95% CI 2.2–120.0), whereas the association was weaker in older patients (HR 1.6, 95% CI 0.94–2.6). Yet, the GLS × age interaction was also non-significant (p interaction = 0.13), suggesting that these apparent differences should be interpreted with caution.

**Supp Figure 1. Survival curves per stage**


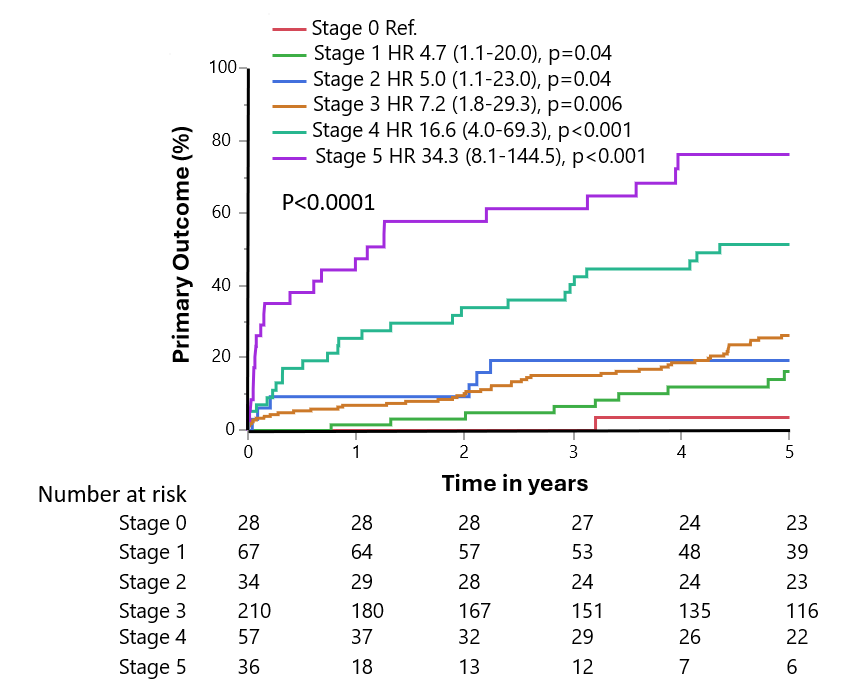

Supplement: Supplementary material [file mmc1.docx]
